# Impact of COVID-19 Among People Who Use Drugs: A Qualitative Study With Harm Reduction Workers and People Who Use Drugs

**DOI:** 10.21203/rs.3.rs-1309886/v1

**Published:** 2022-02-04

**Authors:** Fiona N. Conway, Jake Samora, Katlyn Brinkley, Haelim Jeong, Nina Clinton, Kasey R. Claborn

**Affiliations:** The University of Texas at Austin Steve Hicks School of Social Work; The University of Texas at Austin Steve Hicks School of Social Work; The University of Texas at Austin Steve Hicks School of Social Work; The University of Alabama School of Social Work; Texas Tech University, Department of Psychological Sciences; The University of Texas at Austin Steve Hicks School of Social Work

**Keywords:** COVID-19, pandemic, drug use, harm reduction, people who use drugs, substance use disorder

## Abstract

**Background:**

Fatal drug overdoses in the United States hit historical records during the COVID-19 pandemic. Throughout the pandemic, people who used drugs had greater odds of contracting COVID-19, increased drug use due to COVID-related stress, and heightened levels of anxiety and depression. This exploratory qualitative study examined the specific ways the pandemic negatively impacted people who use drugs.

**Methods:**

Qualitative interviews with 24 people who use drugs and 20 substance use harm reduction workers were conducted. Data from the qualitative interviews were analyzed using applied thematic analysis to identify emergent themes based on the a priori research goals.

**Results:**

Thematic analysis identified several common experiences during the pandemic among people who use drugs. These included mental distress due to financial strain and social isolation; increased drug use; increased risky drug-seeking and use behaviors due to changes in the drug markets; and reduced access to harm reduction, treatment, and recovery support services.

**Conclusions:**

Our study highlighted critical systemic failures that contributed to the rise in overdose deaths during the COVID-19 pandemic. Addressing these challenges through policy reform and improved funding models will ensure the sustainability of harm reduction services and increase access to substance use treatment among highly vulnerable people who use drugs.

## Background

The COVID-19 pandemic is devastating the mental and physical health of millions of people across the globe [1,2]. Everyone is at risk for contracting the illness, but some groups of people experience exponential negative effects due to membership in particularly vulnerable populations. One such group that has been severely neglected throughout the COVID-19 response are people who use drugs [PWUD]. In the United States during 2020, 93,000 people died from drug overdoses—a 30.9% increase from 2019 and the highest change in a 12-month period [3]. Steadily emerging research indicates that poor health outcomes associated with substance use have been particularly amplified during the COVID-19 pandemic. A retrospective study conducted on electronic health records found that individuals with a substance use disorder (SUD) were at much greater odds of contracting COVID-19, and died at a rate 1.5 times greater than those without SUD diagnoses [4]. Death either from drug overdose or from complications of COVID-19 is the most troubling outcome for PUWD during the pandemic, but other consequences of the global health crisis are also important. COVID-related stress has been associated with increases in drug use [5]. This rise in use of illicit substances, including opiates and psychostimulants [6] has contributed to an increase in numerous sequelae associated with SUD. Also, increased loneliness and decreased social connectedness during the pandemic has led to heightened levels of anxiety and depressive symptomatology among PWUD [7].

At the same time that PWUD are experiencing negative health outcomes from the pandemic their access and engagement in treatment has been severely disrupted. Qualitative research about the experiences of PWUD during COVID-19 has been invaluable in providing a lens into their lived experience. One study that provided findings from interviews with directors of residential SUD treatment centers highlighted negative impacts of the pandemic, including less client engagement in treatment, reduced resources and staff, and challenges using telehealth services [8]. Other studies have uncovered that during the pandemic PWUD had exceeding difficulty accessing healthcare-based services (e.g., medication assisted treatment clinics, primary care), and community-based services (e.g., recovery groups) and harm reduction services (e.g., syringe exchange programs, Narcan distribution etc.) [9,10]. In many instances, harm reduction organizations moved to virtual mediums or reduced availability, creating barriers for PWUD in accessing services [11]. Harm reduction services are critical for PWUD who are not engaged in treatment or at the stage of recovery. Services are focused on reducing the harms associated with drug use through programs such as clean needle exchange, naloxone distribution, wound care, safe use education, and other strategies (Beirness et al., 2008; Tatarsky, 2003). It is therefore imperative that harm reduction services be readily available and accessible during a global health crisis.

Recent qualitative studies have made significant contributions to our understanding of the impact of the COVID-19 pandemic on the physical and mental health of PWUD. Almost all of them, however, have only used harm reduction workers (HR) or treatment providers as the source of their information. Thus, their ability to truly capture the experiences of PWUD is limited because their findings are based on secondary sources (providers and service workers), not primary sources (PWUD). None of the studies, to our knowledge, has conducted concurrent interviews of HR and PUWD from the same service area. Our study aimed to address this gap in the literature. We present findings from interviews with both PWUD and HR about the impact of COVID-19 on mental health and substance use among PWUD as well as the impact on harm reduction and treatment services.

## Methods

This exploratory qualitative study examined the specific ways the pandemic negatively impacted people who use drugs. Results from this study are based on a larger parent study designed to understand perspectives on overdose response and prevention efforts in Texas and to develop and implement a digital platform to improve overdose reporting.

### Participants

Participants (n=24 PWUD; n=20 HR) were recruited from four sites in Texas (see Table 1 for participant demographics).

Recruitment methods included flyers, e-mails, telephone calls, snowball sampling, social media advertising, web-postings, word of mouth and use of our Community Advisory Boards. All potential participants were screened with a short survey either via phone call or email by a trained research assistant. The inclusion criteria for HR participants included: 1) eighteen years or older; 2) volunteer or paid staff member for a harm reduction organization; and 3) ability to read and speak in English. The inclusion criteria for PWUD participants included: 1) eighteen years or older; 2) used opioids or stimulants in the past three months, 3) resides in Texas, and 4) ability to read and speak in English. The exclusion criteria for all participants included: the inability or unwillingness to provide consent and being actively intoxicated, suicidal, or psychotic. If a potential participant fit the inclusion criteria, informed consent was provided and obtained.

### Data Collection

Trained research assistants conducted qualitative interviews that lasted 60 to 90 minutes. Participants were compensated $30 for their time. Interviews were conducted by video conference or telephone with two trained researchers. In these interviews we addressed the following research questions: 1) How does COVID-19 impact mental health and substance use among people who use drugs? 2) How does COVID-19 impact harm reduction and treatment services? Participants were assigned a unique ID number and after each interview was completed, the audio recordings were transcribed verbatim by a professional transcription agency. Transcripts were cleaned of all personal identifying information.

### Data Analysis

Data from the qualitative interviews were analyzed using applied thematic analysis, which was selected for its flexibility and systematic approach in analyzing text-based qualitative data [12]. Originally, the team identified emergent themes based on the a priori research goals. Based on these major themes a working codebook and framework matrix was generated. The codebook went through multiple iterations due to emerging data in the transcripts that were relative to our research objectives. Data analysis involved six trained coders (two clinical research associates and four research assistants) by means of a reflexive analysis approach. Each transcript was assigned to two members of the team to code independently using the codebook. Once both members completed their coding process, the members met, while using the reflexive team approach to resolve inconsistencies in coding until they reached a consensus and finalized the coded transcript.

## Results

Thematic analysis of the interviews produced key insights about the mental health and drug use behavior of PWUD during the pandemic. Important themes emerged about (a) increased mental strain, (b) increased drug use, (c) changes in the drug market, and (d) engagement in risky drug seeking and use behavior. Participants also reported difficulty obtaining multiple types of services including (e) treatment, (f) recovery support, and (g) harm reduction. The end of each quote is labeled PWUD or HR to designate which category of participant provided the statement.

### Increased Mental Health Strains

Participants reported experiencing heightened levels of stress due to mounting financial uncertainty with the economic conditions brought on by COVID-19. Some PWUD reported losing jobs, losing housing, and increased debt from borrowing money from friends. Others commented on job loss among family members who they rely on for financial support. One participant noted, *“my dad is in the entertainment industry…but there’s no events right now… he would supplement his income substitute teaching, but schools are closed”* (PWUD). Many made a clear connection between financial challenges and their mental health status. A participant remarked that *“financial stress is the most immediate thing…my mental health has declined”* (PWUD). A harm reduction worker reported their observation of this connection between financial strains and mental distress among PWUD in their service area stating, *“people losing their jobs… it’s caused a lot of stress”* (HR). Some PWUD also relayed that they experienced anxiety about their lack of insurance should they need to be hospitalized due to contracting COVID. In addition, participants reported that social distancing and isolation have also taken a heavy toll on mental well-being. A participant remarked, *“I have anxiety…[I] was diagnosed with depression…it’s made it a lot worse having to stay at home. Not being able to go do things”* (PWUD).

### Increased Drug Use

Participants reported that the pandemic has triggered increased drug use due financial stress, fear of contracting COVID, and boredom due to joblessness and isolation from friends and family. One PWUD remarked *“I definitely have been using drugs more to cope with [stress]. More self-medicating than I have in a long time”* (PWUD). Another noted *“I’m definitely using more…because I don’t really have anything to do all day. I mean, all day I’m just kind of sitting at home”* (PWUD). Some participants reported that people who did not experience joblessness, but transitioned to working at home also increased drug use. One person commented their brother’s drug use saying, *“having to be at home now and working off his computer...he has too much time…[he] just sits there in his house and get[s] high”* (PWUD). Results from interviews with harm reduction workers corroborated reports from PWUD about increased drug use. Workers highlighted the connection between increased drug use and pandemic-related mental health challenges. One of them stated, *“there’s just a lot more use individually, and a lot more use within groups…not having work, being nervous, the anxiety, it’s just leading them to use more than usual”* (HR). Increases in drug use related to COVID-19 was described as highly prevalent from both PWUD and harm reduction workers, illustrating an added challenge to the already disadvantaged position of this vulnerable population.

### Changes in the Drug Market

Themes emerged pertaining to the drug supply chain including reduced supply, increased demand, decreased quality and purity of the drug supply, and a rise in prices of drugs. COVID-19 prompted significant disruptions to the drug supply chain. One explanation for the decrease in availability is that dealers have less supply. A harm reduction worker noted that *“The [drug] supply is dryin’ up…there’s less product out there in the community”* (HR). One reason for the decrease in supply is the reduction in the number of people selling drugs. A worker explained, *“a couple of major drug dealers have already closed shop because they … don’t have access to [suppliers]”* (HR). Additional participants provided other insights about the reasons for the decrease in supply. A PWUD stated, *“well, with the borders [between Mexico and the USA] being shut down, there’s not as much drugs coming across”* (PWUD). One consequence of the disruption in the supply chain was the increase in drug prices. In some instances, both harm reduction workers and PWUD mentioned increased prices for the same type of drug. A harm reduction worker stated, *there’s been crazy spikes in prices, particularly for the methamphetamine, prices have gone up way high. (HR).* This observation was corroborated by a PWUD who reported *“the prices of methamphetamine’s even gone up”* (PWUD). One unexpected outcome of the changes in the drug market was that decreased supply and inflation in prices resulted in decreased consumption for some individuals. One participant reported *“tryin’ to, like, you know, squeeze a whole lot more out of a dose than I normally would”* (PWUD). Another remarked, *“it becomes a point where, you know, it’s not really available to the point where it’s, you know, harder to get than I wanna put effort into”* (PWUD).

### Increased Risky Behaviors

Due to the low supply and increased price of illicit drugs, harm reduction workers expressed added concerns about the increase in risky substance use behaviors. One behavior is switching the drug of choice. One harm reduction worker remarked, *“we’re seeing drugs of choices, and reliable dealers having to be exchanged for questionable substances”* (HR) and a PWUD shared *“I would say it’s changed… the type of drugs I use…cocaine’s expensive and like, ketamine’s expensive as hell”* (PWUD). This highlights how use of specific, more relatively expensive drugs may have decreased, but substance use among individuals increased in possibly riskier ways due to unfamiliarity with different substances. Harm reduction workers also reported that clients in their service areas were engaging in isolated use, putting them at higher risk for fatal overdose. One worker stated, *“A lot of people are using alone because of, social distancing…if something happens… there’s not going to be anybody there to help them out with Narcan [overdose reversal drug] or, like, any other form of resuscitation methods”* (HR). Another worker remarked on hesitation from drug suppliers to allow PWUD into their homes:

“I’ve seen increased paranoia amongst [dealers], as well, where they’re hesitant to allow people into their apartments. They’re hesitant to allow people into their area because they don’t know if they’ve been infected [with COVID]...[before the pandemic] a lot of the overdose reversals I see taking place, happen in the supplier’s home but one person, in particular, is like, ‘I don’t really know if I want people, like, hanging out here right now.’ And we know that means there’s an increased likelihood of them…using in solitude, by themselves.”

(HR, 108).

The impact on safe use practices by COVID-19’s social distancing and quarantine requirements adds to the potential risk of overdose, exacerbating the already present risks of overdose when using substances. In addition to the risks associated with switching drugs, and using drugs alone, workers also expressed concerns about increased risky drug-seeking behaviors. Multiple harm reduction workers shared concerns that people in their service area could be compromising their safety to obtain drugs. One worker shared, *“[PWUD] have to take riskier chances to get money...you can’t panhandle like a lot of people do…there’s just nobody out on the streets [because of COVID] …so you’re seein’, uh, riskier behaviors”* (HR). Another noted the dangers of PWUD seeking drugs in locations outside of their usual areas remarking, *“they’re gonna go outside their community to get what they need, you know, and that puts them at higher risk of not only, you know, COVID-19, but also police persecution, [and] overdose”* (HR).

### Reduced Access to Treatment and Recovery Support

COVID-19 has an undeniable impact on the mental health and substance use patterns of PWUD and the same is true for harm reduction, treatment, and recovery support services. Participants shared that there was an increased demand for services. A harm reduction worker reported:

Some people who I’ve talked to recently have reported almost wanting to… try to get clean during this time… may as well try to find a detox facility right now or find medication assisted treatment, because either the [drug] prices are so high, or it’s so difficult to obtain.

(HR)

Although demand for services increased, participants reported experiences of disruptions and reduced access to providers and resources, including phone lines being rerouted, programs shutting down, and clinics being unresponsive. One participant who was enrolled in a methadone program described their difficulty reaching a caseworker expressing; *“now they’ve changed his number where I can’t call him...I would call him like once a week and tell him how I was…how I’m supposed to get in touch with him? I don’t like it at all”* (PWUD). This lack or access also affected PWUD who needed recovery support services. Participants indicated that the pandemic had *“pretty much stopped AA meetings around, you know, everywhere [making it] kinda difficult for people who are in recovery…[to]maintain sobriety, you know?”* (PWUD). As PWUD rely on resources such as support groups and rehab/detox services to sustain recovery, these pandemic-related frustrations caused major disruptions to their recovery path. One participant described the difficulty of getting treatment for a friend: *“We tried to get him into a rehab…anybody that we could get to answer the phones, they were like, yeah, we don’t know what’s going on, just because of this whole COVID thing. We’re not getting anybody in anywhere right now.”* (PWUD).

### Reduced Access to Harm Reduction Services

In addition to lack or disruption of treatment and recovery services, PWUD reported on the diminished availability of harm reduction services. One participant remarked on the lack of access to these services and the resources provided, saying that they *“went to a [homeless] camp that’s primarily heroin use…[people]said that no [harm reduction worker has] been out there for months. They don’t have access to syringes or anything or Narcan”* (PWUD). Another participant who disclosed that they were engaged in harm reduction services provided by the same organization for over two year remarked:

[The organization] had a van that would go and meet people five days a week at two locations…but once all this [i.e., the pandemic] started… they immediately cut the five days off, and I think there was a week or two that they didn’t do anything.

(PWUD)

Harm reduction workers also shared their experiences and frustrations with their diminished capacity to provide services. They reported rapid and drastic changes in their provision of services and in systemic policy changes due to public health safety measures in response to the pandemic, including relocating service sites, reducing operating hours, suspending services, and stopping face-to-face interactions. One worker remarked, *“I cannot go out into the field. I haven’t done outreach…I haven’t seen my patients”* (HR). Mitigation of in-person contact was especially damaging to service provision for people living without shelter or homes. One worker shared that before the pandemic workers *“used to just go into the [homeless] encampments and talk to everybody that [they] could and now…going into the homeless camp and having a seat and a discussion with folk…that’s no longer happening”* (HR). Harm reduction workers also shared that there was negative response in some of their communities regarding their field work. One worker explained:

[We]encountered [opposition] from some of the communities where we normally set up because there’s an increased sensitivity to seeing, all of a sudden, large crowds of people with limited access to PPE [personal protective equipment] hovering in areas, in neighborhoods.

(HR)

Another worker expressed frustration at systemic changes stemming from competing and often conflicting guidelines from local and state public health agencies saying, *“I think that a lot of harm reduction organizations like mine were really impacted by statewide orders that superseded local [orders]”* (HR). Harm reduction services being difficult to access despite high demand, increased risky use behaviors. Health and safety for injection drug users were at greater risk. One participant reported, *“trying to make [syringes] last longer, and the longer I use ‘em, the duller they get, and the more that it ends up really hurting, and it ends up like tearing, you know”* (PWUD). Although the pandemic negatively impacted the availability and quality of harm reduction services, one worker viewed the need for rapid organizational change as an opportunity for positive public policy changes stating:

There’s some major regulatory changes…we’re having to coordinate in ways that we never thought of before, and I think what’s gonna come as a result of this is a realization that a lot of the regulations that have been in place actually are unnecessary and actually are contributing to mortality.

(HR)

Reduced services that resulted from COVID-19 increased risky behaviors and has drawn attention to the importance of harm reduction work in local communities. Despite the organizational and systemic changes caused by the pandemic, many harm reduction worker remained steadfastly committed to providing services to people in their communities. One worker said:

“A lot of [staff] are still willing to, you know, go in every day and do the work that needs to be done. Because people in harm reduction are very passionate about what they do…the pandemic is a new situation for sure, but, you know, we’ve done it…whether or not it’s safe to do sometimes. Like lines [i.e., COVID safety guidelines] have been crossed for sure. But I think that’s just because, the drive, and the passion.”

(HR).

## Discussion

Fatal overdoses in the United States hit historic records during the COVID-19 pandemic and the healthcare system failed to adequately support the needs of people who use drugs during this global crisis. During the past two years there have been major advancements in the availability of protective vaccines and some people are feeling less isolated due to loosened official restrictions on social contacts. Still many COVID-related stressors remain. Unemployment is a persistent issue and many people are still working from home part-time or full-time. In addition fear of contracting COVID due to variants—such as Delta and Omicron—remains. This fear can cause increased anxiety and influence many people to self-isolate despite changes to government policies or recommendations from the Center for Disease Control. Understanding factors associated with COVID-related riskier drug seeking and use behaviors, as well as understanding pandemic-related stressors that contribute to overdose risk remains a critical area of study towards developing strategies to mitigate the negative sequelae of the COVID-19 pandemic among people who use drugs.

Our study also highlighted critical systemic failures that have contributed to the rise on overdose deaths. Changes in the drug market reduced supply and decreased the quality and purity of the drug supply. People who use drugs were forced to obtain drugs from unknown suppliers as their previous supplier may not have had access to drugs or were engaging in social distancing practices. Further, the fragility of the mental health and substance use prevention and treatment infrastructure was exemplified through the closure or reduction in services. Vulnerable populations with limited access to internet or reliable cell phone data plans were disadvantaged and lacked the ability to access telehealth service options. The negative economic impact on these prevention and treatment organizations, particularly those serving the most vulnerable populations, resulted in many organizations permanently closing or suspending services during the pandemic. This demonstrates the need for improved funding models to sustain these critical services. Ensuring uninterrupted harm reduction, treatment, and recovery services, particularly during a pandemic requiring social isolation at a time of immense uncertainty and stress, is a critical public health strategy to prevent fatal overdoses.

### Limitations

These findings should be taken in light of several limitations. First, this study was qualitative in nature. As such, our sample included people who use drugs and harm reduction workers located in Texas. The political infrastructure and the fact that Texas borders Mexico may limit the generalizability of these findings to other geographic locations. Second, future studies should seek to quantitatively examine the factors identified through our qualitative methods to inform frequency and impact among this population. Finally, our sample was primarily White and heterosexual, limiting extrapolation of findings to sexual orientation and gender minority groups and other racial/ethnic populations.

## Conclusion

This exploratory qualitative study identified key factors that should be considered in disaster planning and risk mitigation strategies for future pandemics. More importantly, it highlighted key infrastructure challenges inherent in the addiction prevention and treatment continuum. These challenges need to be addressed through policy reform and improving funding models to improve sustainability of harm reduction services and increase access to substance use treatment among highly vulnerable people who use drugs.

## Figures and Tables

**Table 1.** Participant Characteristics

	Harm Reductionists (n=20)		People Who Use Drugs (n=24)	
	*n*	%	*n*	%
**Age**				
Median (IQR)	35	(29–50)	36	(29–43)
**Gender at birth**				
Female	10	50	13	54.2
Male	10	50	11	45.8
Intersex	-	-	-	-
**Gender Identity**				
Male	9	45	13	54.2
Female	9	45	11	45.8
Transgender (FTM)	1	5	-	-
Transgender (MTF)	-	-	-	-
Non-Binary	1	5	-	-
Other	-	-	-	-
Unknown	-	-	-	-
Prefer not to answer	-	-	-	-
**Sexual Orientation**				
Gay/Lesbian	2	10	1	4.2
Straight/Heterosexual	11	55	21	87.5
Bisexual	5	25	2	8.3
Other	3	15	-	-
Prefer not to answer	-	-	-	-
**Race**				
Asian	2	10	-	-
American Indian/Alaskan Native	1	5	-	-
Black/African American	2	10	1	4.2
Pacific Islander/Native Hawaiian	-	-	-	-
White/Caucasian	14	70	20	83.3
Other	4	20	5	20.8
**Ethnicity**				
Hispanic or Latino	8	40	10	41.7
Not Hispanic or Latino	11	55	13	54.2
Prefer not to answer	1	5	1	4.2

## Abbreviations

PWUD: People Who Use Drugs
HR: Harm Reduction Worker
